# Return to physical activity and change in body mass index after hypoallergenic medial mobile-bearing unicompartmental knee arthroplasty

**DOI:** 10.1186/s10195-021-00598-4

**Published:** 2021-09-18

**Authors:** Riccardo D’Ambrosi, Alessandro Nuara, Ilaria Mariani, Katia Corona, Stefan Mogos, Francesco Catellani, Michael Hantes, Nicola Ursino

**Affiliations:** 1 IRCCS Istituto Ortopedico Galeazzi, Milan, Italy; 2 Dipartimento Di Scienze Biomediche Per La Salute, Università Degli Studi Di Milano, Milan, Italy; 3 Institute for Maternal and Child Health-IRCCS “Burlo Garofolo”, Trieste, Italy; 4 Dipartimento Di Medicina E Scienze Della Salute Vincenzo Tiberio, Università Degli Studi del Molise, Campobasso, Italy; 5 Foișor Orthopaedics Hospital, Bucharest, Romania; 6 Humanitas Gavazzeni, Bergamo, Italy; 7 Department of Orthopaedic Surgery, Faculty of Medicine, University of Thessalia, University Hospital of Larissa, Larissa, Greece

**Keywords:** Hypoallergenic arthroplasty, Knee osteoarthritis, Return to sport, Unicompartmental knee arthroplasty

## Abstract

**Background:**

The primary purpose of the present prospective study was to consecutively analyse the outcomes of the return to sports activity of patients with positive patch tests undergoing a medial mobile-bearing titanium niobium nitride (TiNbN) unicompartmental knee arthroplasty (UKA). The secondary purpose was to ascertain if a higher grade of physical activity leads to a reduction in the body mass index (BMI) of the patients.

**Material and methods:**

Forty-one patients with positive skin patch tests were included in this prospective study. The clinical evaluation consisted of the University of California, Los Angeles (UCLA) activity scale and the High-Activity Arthroplasty Score (HAAS). Each patient was evaluated the day before surgery (*T*_0_), after 12.37 ± 0.70 months (*T*_1_), and on the day of the final follow-up, after 67.03 ± 18.2 months (*T*_2_). Furthermore, the BMI of each patient was analysed before surgery and during the final follow-up.

**Results:**

The UCLA and HAAS mean preoperative values ranged from 3.68 ± 1.1.7 and 6.15 ± 0.76 to 6.1 ± 0.76 and 10.34 ± 1.3, respectively, at *T*_1_ (*p* < 0.0001) and to the final values of 6.34 ± 0.62 and 11.0 ± 8.9, respectively, at *T*_2_ (UCLA: *T*_2_ versus *T*_1_: *p* = 0.132; T_2_ versus *T*_0_: *p* < 0.0001; HAAS: *T*_2_ versus *T*_1_: *p* = 0.0027; *T*_2_ versus *T*_0_: *p* < 0.001). BMI ranged from a preoperative value of 27.97 ± 3.63 to a final value of 26.84 ± 3.11 (*p* < 0.0001). The only differences within the subgroups concerned patients with BMI ≥ 28, showing a superior HAAS at each follow-up (*p* < 0.05). A positive correlation was found between BMI and HAAS at *T*_0_ and *T*_2_ (*p* < 0.05).

**Conclusions:**

This is the first study to evaluate the rate of the return to sports activities and change in BMI following hypoallergenic UKA. The majority of patients reduced their weight following UKA and improved their physical activity, showing outcomes that were comparable to the standard cobalt–chrome (CoCr) prostheses, regardless of gender, age, BMI and implant size.

**Level of evidence:**

IV – Prospective Cohort Study.

*Trial registration* researchregistry5978—Research Registry www.researchregistry.com

**Supplementary Information:**

The online version contains supplementary material available at 10.1186/s10195-021-00598-4.

## Introduction

In the past two decades, a renewed interest was noted in unicompartmental knee arthroplasty (UKA) for the treatment of isolated knee arthritis, especially in young and active patients [1]. In such patients, problems such as functional recovery and the possibility of returning to sports should be considered. The rate of return to sports following UKA ranges from 87% to 97% [2]. It is documented that patients undergoing UKA return to sports and work faster with better postoperative knee scores than those undergoing total knee implants [3, 4]. The main reason for these reports could lie in the low invasiveness of the surgical technique and the preservation of the cruciate ligaments. As a matter of fact, the restoration of the physiological knee function, in addition to a short hospital stay, may contribute to explaining the high return rate to sports [5]. The patients who preoperatively participate in low-impact sports achieve the highest level of satisfaction after knee replacement. Consequently, the most practised sports following surgery are hiking, cycling and swimming. On the other hand, there is a postoperative reduction in high-impact sports such as football, tennis and skiing [6–8]. In view of the considerable increase in the use of UKA, prostheses of hypoallergenic materials have been developed. These materials provide an excellent alternative to the standard cobalt–chrome (CoCr), especially in patients who have metal hypersensitivity, one of the most controversial issues as a cause of premature failure in knee arthroplasty [9, 10]. Ceramic coatings have been proposed since they are chemically inert, with high hardness and bioactive features. Titanium niobium nitride (TiNbN) deposited by a physical vapour deposition (PVD) technique is a thin ceramic coating characterised by an increase in the surface’s resistance to abrasion and corrosion, thus acting on several biological processes with a possible improvement in the biocompatibility and osteointegration of the implants in the long term. TiNbN coating acts as a surface isolating layer to hide the metallic device beneath from the biological environment [11]. To date, there are no clinical studies reporting outcomes of the return to sports in patients who underwent anallergic TiNbN medial UKA. The primary purpose of the present prospective study is to consecutively analyse the outcomes of the return to sports activity in patients with positive patch tests undergoing a medial mobile-bearing TiNbN UKA during their mid-term follow-ups. The secondary purpose is to evaluate if a higher grade of physical activity leads to a reduction in the body mass index (BMI) of the patients.

## Material and methods

All the procedures involving human participants in this study were in accordance with the ethical standards of the institutional and/or national research committee, as well as the 1964 Helsinki Declaration and its later amendments or comparable ethical standards. The study was conducted following the STROBE checklist for case series and registered in the Research Registry (researchregistry5978; www.researchregistry.com) [12]. Informed consent was obtained from all the participants who were included in the study. Appropriate ethical approval was obtained from the local ethics committee. A consecutive series of 472 patients suitable for medial UKA were screened for metal hypersensitivity using a standardised questionnaire and, in case of suspicion, a patch test. The reactions were scored using the International Contact Dermatitis Research Group (ICDRG) criteria [13]. A total of 41 patients (8.68%) with a positive epicutaneous patch test result for metals such as nickel, cobalt and chromium were included in this study and prospectively monitored. The demographics are summarised in Table 1. All the patients underwent the UKA with the same type of implant – a cemented TiNbN hypoallergenic medial UKA Oxford (Zimmer-Biomet, Warsaw, Indiana, USA) with a mobile-bearing inlay.

**Table 1:** Demographic data of the patients included in the study

Variables	*N* = 41
Gender	
Male	8 (19.5)
Female	33 (80.5)
Side	
Left	18 (43.9)
Right	23 (56.1)
*T*_1_ (months)	12.37 ± 0.70
*T*_2_ (months)	67.03 ± 18.62
Age at *T*_0_	66.41 ± 6.50
Weight (kg)	73.17 ± 10.82
Height (m)	1.62 ± 0.04

Values are presented as *n* (%) or mean ± SD

The primary indication for surgery was isolated osteoarthritis (OA) in the medial knee compartment with full cartilage loss (‘bone-on-bone articulation’) or avascular necrosis of the femoral condyle. In all the cases, the anterior cruciate, medial collateral and lateral collateral ligaments were functionally intact, as confirmed by magnetic resonance imaging (MRI), and the varus or valgus deformity was manually correctable. There was no evidence of OA in the lateral compartment. Rheumatoid arthritis, fixed varus/valgus deformities, previous osteotomy and flexion deformities greater than 15° were considered as contraindications, in accordance with the reported Oxford criteria [14].

### Surgical technique

All the operations were performed by a senior surgeon experienced in UKA arthroplasty (for more than 20 years) (NU) using a minimally invasive surgical technique. It involved a medial parapatellar approach without the dislocation of the patella using microplasty instrumentation. Immediate full weight-bearing was postoperatively allowed [15, 16].

### Clinical evaluation

All the clinical assessments were performed by an independent clinician who was not involved in the index surgery. The clinical evaluation consisted of evaluating each patient’s University of California, Los Angeles (UCLA) activity scores and the High-Activity Arthroplasty Score (HAAS). Each patient was clinically evaluated on the day before surgery (*T*_0_), after 12.37 ± 0.70 months (*T*_1_), and on the day of the final follow-up, after 67.03 ± 18.62 months (*T*_2_) [17, 18]. Finally, the BMI of each patient was measured before surgery (*T*_0_) and at the final follow-up (*T*_2_: 67.03 ± 18.62 months) [19].

### Radiographic assessment

The radiograph evaluations were performed blindly by two orthopaedic surgeons not involved in the index surgery. The positioning of the UKA was evaluated using radiological analysis during the final follow-up (*T*_2_: 67.03 ± 18.62 months), as specified in the Oxford Partial Knee Surgical Technique operating manual, using standing anteroposterior (AP) and lateral radiographs of the knee. The following parameters were measured according to the manufacturer’s manuals [6]: the femoral component varus/valgus, i.e. the angle between the femoral component and femoral axis in the coronal plane, for which an angle of 7° was seen as neutral with a range of tolerance of ±10°; the tibial component varus/valgus, i.e. the angle formed by the tibial axis and a line drawn along the tibial tray in the coronal plane, for which the range of tolerance was 0° ± 5°; finally, the anteroposterior slope, i.e. the angle of a line drawn along the tibial tray and perpendicular to the tibial axis in the lateral view, for which a slope of 7° was seen as optimal with a range of tolerance of ± 5° (2–12°) [6].

After the Digital Imaging and Communications in Medicine (DICOM, Arlington, Virginia, USA), data were extracted from the Picture Archiving and Communications System (PACS, Carestream, Rochester, New York, USA) and inserted into the OsiriX imaging software (version 4.1.2 32-bit) (Pixmeo SARL, Geneva, Switzerland) and evaluated by two independent observers who were unaware of the instrumentation used for UKA.

### Statistical analysis

An estimated sample size of 35 subjects was calculated using a one-tailed paired *t*-test to detect a 3-point increase in both UCLA and HAAS scores in subsequent time points, assuming a standard deviation of 4, a two-sided alpha of 5% and a power of 99%. Data of the patients included in the study are reported in Additional file 1. Moreover, six additional subjects were recruited to ensure statistical significance in the case of unexpected events. Data were presented as frequencies with respective proportions for categorical variables and as mean and standard deviation (SD) for continuous variables. Firstly, to assess variation in time (preoperative; *T*_0_) and first (*T*_1_) and second follow-up visits (*T*_2_) of UCLA and HAAS scores, a mixed model was performed using an unstructured covariance matrix for repeated measurements on subjects. Secondly, a subgroup analysis was conducted by gender, age and obesity (BMI < 30 or BMI ≥ 30). Age groups were defined after dichotomising the age at its average rounded value. Score means of different groups were compared at the same time period (within-time comparison) and score means of the same group were compared at different time periods (within-group comparison) using mixed models. A within-time comparison was also performed for the slope, femoral and tibial angle with a *t*-test. Bonferroni correction was used for multiple comparisons. Thirdly, the correlation among scores, BMI and angles was estimated and tested. In the case of deviation from the assumptions of models and tests, an equivalent non-parametric test (from among the Wilcoxon signed-rank, Wilcoxon–Mann Whitney and Spearman rank correlation tests) was performed. A two-tailed *p*-value of less than 0.05 was considered statistically significant. Statistical analyses were performed using SAS System Version 9.

## Results

The UCLA and HAAS mean preoperative values ranged from 3.68 ± 1.17 and 6.15 ± 0.76 to 6.15 ± 0.76 and 10.34 ± 1.3, respectively, at *T*_1_ (*p* < 0.0001), and to the final values of 6.34 ± 0.62 and 11.0 ± 8.9, respectively, at *T*_2_ (UCLA: *T*_2_ versus *T*_1_: *p* = 0.132; *T*_2_ versus *T*_0_: *p* < 0.0001; HAAS: *T*_2_ versus *T*_1_: *p* = 0.0027; *T*_2_ versus *T*_0_: *p* < 0.001). BMI ranged from a preoperative value of 27.97 ± 3.63 to a final value of 26.84 ± 3.11 (*p* < 0.0001). At T_2_, the mean femoral angle was 6.90° ± 4.27°, the mean tibial angle was 2.83° ± 2.07° and the mean tibial slope was 4.90° ± 3.10°. Detailed results are reported in Table 2. All the analysed subgroups showed significant improvements between the preoperative value (*T*_0_) and the final follow-up (*T*_2_) (*p* < 0.0001). The only differences within the subgroups concerned patients with BMI ≥ 28, showing a superior HAAS at each follow-up (*p* < 0.05). Detailed results are reported in Table 3. No radiographic differences were found among subgroups (*p* > 0.05). Detailed results are reported in Table 4. A positive correlation was found between BMI and HAAS at *T*_0_ and *T*_2_ (*T*_0_: rho = 0.52606, *p* = 0.0004; *T*_2_: rho = 0.31291, *p* = 0.0464).

**Table 2:** Clinical and radiographic outcomes and BMI at different follow-ups and the relative *p*-value

	*T*_0_	*T*_1_	*T*_2_	Mean difference (adjusted *p*-value)
BMI	27.97 ± 3.63	–	26.84 ± 3.11	*T*_2_–*T*_0_: < 0.0001*
UCLA	3.68 ± 1.17	6.15 ± 0.76	6.34 ± 0.62	*T*_1_–*T*_0_: < 0.0001* *T*_2_–*T*_0_: < 0.0001* *T*_2_–*T*_1_: 0.1321
HAAS	6.15 ± 0.76	10.34 ± 1.30	11 ± 0.89	*T*_1_–*T*_0_: < 0.0001* *T*_2_–*T*_0_: < 0.0001* *T*_2_–*T*_1_: 0.0027*

Values are presented as mean ± SD

*n.a.* not available

* Statistically significant difference

**Table 3:** Clinical comparisons between groups divided on the basis of gender, age and BMI

Score	Time period	Groups		Mean score comparison within time period	Mean score comparison within groups (Bonferroni-adjusted *p*-value)		
		Gender		*p*-Value		Gender	
		Male (*n* = 8)	Female (*n* = 33)			Male (*n* = 8)	Female (*n* = 33)
UCLA	*T*_0_	3.75 ± 1.67	3.67 ± 1.05	0.8594	*T*_1_–*T*_0_:	0.0002*	< 0.0001*
	*T*_1_	6.13 ± 0.35	6.15 ± 0.83	0.9308	*T*_2_–*T*_0_:	< 0.0001*	< 0.0001*
	*T*_2_	6.13 ± 0.35	6.39 ± 0.66	0.2740	*T*_2_–*T*_1_:	1.000	0.0771
HAAS	*T*_0_	4.38 ± 1.92	4.55 ± 1.54	0.7906	*T*_1_–*T*_0_:	< 0.0001*	< 0.0001*
	*T*_1_	10.50 ± 1.07	10.30 ± 1.36	0.7050	*T*_2_–*T*_0_:	< 0.0001*	< 0.0001*
	*T*_2_	11.13 ± 0.99	10.97 ± 0.88	0.6652	*T*_2_–*T*_1_:	0.4361	0.0078*

		Age		*p*-Value		Age	
		< 65 years (*n* = 17)	≥ 65 years (*n* = 24)			< 65 years (*n* = 17)	≥ 65 years (*n* = 24)
UCLA	*T*_0_	3.41 ± 0.62	3.88 ± 1.42	0.1175	*T*_1_–*T*_0_:	< 0.0001*	< 0.0001*
	*T*_1_	6.18 ± 0.95	6.13 ± 0.61	0.5446	*T*_2_–*T*_0_:	< 0.0001*	< 0.0001*
	*T*_2_	6.47 ± 0.72	6.25 ± 0.53	0.1629	*T*_2_–*T*_1_:	1.000	0.7500
HAAS	*T*_0_	4.24 ± 0.66	4.71 ± 2.01	0.3572	*T*_1_–*T*_0_:	< 0.0001*	< 0.0001*
	*T*_1_	10.71 ± 1.40	10.08 ± 1.18	0.1315	*T*_2_–*T*_0_:	< 0.0001*	< 0.0001*
	*T*_2_	11.18 ± 1.01	10.88 ± 0.80	0.2934	*T*_2_–*T*_1_:	0.3234	0.0064*

		BMI		*p*-Value		BMI	
		0< 30 (*n* = 24)	≥ 30 (*n* = 17)			< 30 (*n* = 24)	≥ 30 (*n* = 17)
UCLA	*T*_0_	3.83 ± 1.01	3.47 ± 1.37	0.3946	*T*_1_–*T*_0_:	< 0.0001*	0.0007*
	*T*_1_	6.17 ± 0.64	6.12 ± 0.93	0.9081	*T*_2_–*T*_0_:	< 0.0001*	< 0.0001*
	*T*_2_	6.29 ± 0.62	6.41 ± 0.62	0.5330	*T*_2_–*T*_1_:	1.000	0.7500
HAAS	*T*_0_	4.08 ± 1.67	5.12 ± 1.32	0.0396*	*T*_1_–*T*_0_:	< 0.0001*	< 0.0001*
	*T*_1_	9.96 ± 1.20	10.88 ± 1.27	0.0225*	*T*_2_–*T*_0_:	< 0.0001*	< 0.0001*
	*T*_2_	10.75 ± 0.79	11.35 ± 0.93	0.0316*	T_2_–T_1_:	0.0001*	< 0.0001*

Values are presented as unadjusted mean ± SD

* Statistically significant difference

**Table 4:** Correlations among sports activities and BMI

Variable	BMI at *T*_0_		BMI at *T*_2_	
	Rho	*p*-Value	Rho	*p*-Value
UCLA at *T*_0_	−0.11226	0.4847	−0.10449	0.5156
UCLA at *T*_1_	−0.17627	0.2703	−0.00395	0.9805
UCLA at *T*_2_	−0.05817	0.7179	0.01634	0.9192
HAAS at *T*_0_	0.52606	0.0004*	0.46529	0.0022*
HAAS at *T*_1_	0.25564	0.1067	0.25339	0.1099
HAAS at *T*_2_	0.29199	0.0640	0.31291	0.0464*

* Statistically significant difference

## Discussion

The most important findings of the present study have been presented as follows: in selected patients with a positive epicutaneous patch test result, medial mobile-bearing TiNbN UKA allows a return to sports and physical activities in the medium term without any complications, regardless of age, gender or weight. Furthermore, a significant improvement in BMI was noted in these patients, probably due to increased sports activities. Finally, even if the TiNbN femoral component presented only one femoral peg, no signs of mobilisation or subsidence were noted during the follow-ups.

To our knowledge, this is the first study reporting return to sports in patients who underwent hypoallergenic TiNbN mobile-bearing UKA. Allergy in knee arthroplasties is an issue that has become increasingly prominent over the last few years, with the number of patients undergoing primary knee arthroplasty increasing annually and between 10% and 48% of the population being sensitive to metal, most commonly nickel [9, 10]. Metallic implants are used widely in surgery, including orthopaedic, cardiothoracic, vascular and maxillofacial surgery, and dentistry. While there are many individual case reports of localised and systemic effects related to metal implants, there appears to be a lack of strong evidence to support or refute the hypothesis that they are caused by allergy [20].

Recently D’Ambrosi et al. evaluated 37 patients with metal hypersensitivity, showing good to excellent clinical and radiographic results at a medium follow-up without complications using a TiNbN medial mobile-bearing UKA [21]. Moreover, Walker et al. demonstrated no local or systemic symptoms of metal hypersensitivity in 82 patients reporting signs of metal hypersensitivity using a standard CoCr [6]. In fact, at a mean follow-up of 3 years, only one patient underwent revision surgery to a bicondylar prosthesis owing to a tibial periprosthetic fracture, resulting in a survival rate of 98.8% with the endpoint of revision for any reason and a survival rate of 97.6% for the endpoint of all reoperations. The clinical outcome was good to excellent with a mean Oxford Knee Score of 42.5 [6]. Furthermore, in 2018, Thomas et al. compared two groups of total knee arthroplasty (TKA) patients with a 5-year follow-up who had had identical types of prosthesis with one deviation: in half of the patients, a variant was implanted with multilayer advanced surface (AS) coating with zirconium nitride (ZrN) to reduce metal ion release, assessing the clinical performance of the two variants of TKA in these patients and the immune reactivity to TKA, with a focus on the systemic response [22]. On 5-year follow-up, the survival rate was 98% for coated and 97% for uncoated implants. The mechanical axis and pain score was comparable. Most serum cytokine levels were comparable, but mean interleukin-8 and interleukin-10 levels were higher in the group with an uncoated implant [22].

Over the last years, significant improvements have been reported in UKA design concepts, leading to excellent clinical results and a higher rate of survivorship [23]. Consequently, UKAs are increasingly being used in younger and more active patients [24]. This particular patient cohort, however, has high expectations concerning the post-operative level of physical activity. Return to activity following UKA – be it sports or other activities – is of concern to every patient. It is well known that engaging in physical activity from low to moderate intensity is safe, thereby increasing standards of living through higher physical and social mobility and better cardiovascular performance [25]. Furthermore, and from a public health perspective, this reduces costs related to treatments in this population. Some authors have even reported the benefits of knee arthroplasty in general health and sports performance [23–25]. However, returning to sports activity after knee arthroplasty is not as thoroughly studied as other aspects of functional recovery.

In the current study, we demonstrated a considerable return to sports activities in patients who underwent hypoallergenic UKA with a consequential BMI decrease. A systematic review published in 2016 supports our results; in fact, Waldstein et al. analysing contemporary literature found in ten studies a statistically significant improvement of physical activity following UKA according to the UCLA activity score, the Tegner activity score and the HAAS [2]. Hiking, cycling and swimming are the most common activities following UKA. Sports participation before the onset of restricting symptoms ranged from 64% to 93% and slightly decreased by 2% to 9% following UKA. The rate of return to activity ranged from 87% to 98% [2].

Similar results were found by Lo Presti et al., who analysed 53 athletic patients who underwent cemented medial UKA. At the final follow-up of 48 months, 48 out of 53 patients were engaged in sports and recreational disciplines, resulting in a return to activity rate of 90% [5]. No early failure and no cases of revision were reported. The frequency of activities (sessions per week) and the periods of time remained constant at the time of the survey. The most common activities after surgery were hiking, cycling and swimming. Several high-impact activities, including skiing and football, significantly decreased among participating patients [5]. Furthermore, UKA can be considered a winning choice in elderly active patients, as reported by Papalia et al. and as confirmed in a recent systematic review in which the rate of return to sports in elderly patients after UKA was 86%, also showing a better relative return to sports and time taken to return for patients undergoing UKA compared with those undergoing TKA [26]. Higher rates of sport‐specific return were observed for low-impact sports, whereas a full return to activities was prevented in high‐impact sports. With regard to the return to sports after UKA, Harbourne et al. evaluated predictive factors of return to desired activities 12 months after surgery; worse Oxford Knee Score, higher BMI and worse expectations were identified as negative predictive factors [27].

Finally, we analysed how the BMI varies at the final follow-up and shows a significant decrease, also directly linked to the increase in sports activities.

These data have not been analysed in the literature, but this study shows how physical activity has a systemic impact. Previous studies have focused more on weight as a possible positive or negative predictive factor in UKA. A recent retrospective multi-centre study analysed the data of patients who received unicompartmental knee prosthesis in order to examine if obesity affects clinical outcomes; the conclusion was that obese and morbidly obese patients have as much to gain from total knee replacement as non-obese patients [28]. Sundaram et al., in 2019, assessed the influence of BMI on 30-day postoperative complications after UKA when analysed as both a categorical and continuous variable, highlighting that overweight and obese individuals who undergo UKA may not have an increased risk of 30-day postoperative complications compared with normal-weight individuals [29].

In 2017, Xia et al. performed a study similar to ours, evaluating the prevalence of patients who lost or gained weight following UKA, the effect of post-operative BMI changes on functional outcomes and quality of life (QoL) and predictive factors associated with BMI changes. Following UKA, 138 (13.3%) patients lost weight, 695 (66.6%) maintained their weight and 210 (20.1%) gained weight. Patients in all groups demonstrated significant improvements in functional and physical quality of life component (SF-36 PCS) scores post-operatively at 2 years. There were no significant differences in functional outcome, QoL or revision rate between the groups. Post-operative BMI changes were not correlated with any outcome scores [30]

This study has some limitations. Firstly, the study sample was small; however, a power analysis was accurately performed to gain a significant cohort with strict inclusion criteria. Secondly, no control group was reported, and no comparison with current literature was performed due to the lack of similar articles. Thirdly, the results cannot be generalised as only one specific mobile-bearing UKA was used. Lastly, radiographic scores were evaluated only at final follow-up.

## Conclusions

This is the first study to evaluate the return to sports activities and change in BMI following hypoallergenic UKA. The majority of patients reduced their weight following UKA and improved their physical activity; the outcomes were comparable with the standard CoCr prostheses, regardless of gender, age, BMI and implant size, among metal allergy patients with medial knee osteoarthritis. These findings will provide important information to surgeons when counselling patients regarding BMI change and its effect on outcomes after UKA (Additional file 1).

## Supplementary Information

**Additional file 1. **Dataset of the patients included in the study.

## Data Availability

Dataset has been included as Additional file 1.
